# Experimental Use of Common Marmosets (Callithrix jacchus) in Preclinical Trials of Antiviral Vaccines

**DOI:** 10.32607/actanaturae.27372

**Published:** 2024

**Authors:** I. V. Gordeychuk, O. S. Gancharova, S. A. Gulyaev, T. V. Gulyaeva, A. S. Zhitkevich, D. V. Avdoshina, A. V. Moroz, A. S. Lunin, S. E. Sotskova, E. A. Korduban, A. I. Tukhvatulin, E. O. Bayurova, A. A. Ishmukhametov

**Affiliations:** Chumakov Federal Scientific Center for Research and Development of Immune-and-Biological Products of Russian Academy of Sciences, Moscow, 108819 Russian Federation; Institute for Translational Medicine and Biotechnology, Sechenov University, Moscow, 117418 Russian Federation; Belozersky Institute of Physico-Chemical Biology, M.V. Lomonosov Moscow State University, Moscow, 119992 Russian Federation; National Research Centre for Epidemiology and Microbiology named after Honorary Academician N.F. Gamaleya of the Ministry of Health of the Russian Federation, Moscow, 123098 Russin Federation

**Keywords:** laboratory primates, Callithrix jacchus, laboratory breeding of primates, antiviral vaccines, safety and immunogenicity of vaccines

## Abstract

Common marmoset (*Callithrix jacchus*, CM) is a New World primate species that is of interest for preclinical trials of immunobiological products. In this study, we describe the approaches to long-term laboratory breeding and maintenance of CMs. We also establish the reference values of the main complete blood count and serum chemistry parameters evaluated during preclinical trials of immunobiological products and describe the histological characteristics of CM lymphoid organs during the development of post-vaccination immune response. We show that CMs bred in laboratory conditions excluding background infectious pathology are a relevant model that allows for a high degree of reliability in characterizing the safety and immunogenicity profile of antiviral vaccines during preclinical trials.

## INTRODUCTION

Nonhuman primates are the most suitable laboratory model for most human viral diseases. They allow for adequate reproduction of the stages of development of viral infections, including the route of transmission, the virus replication site, the pathogenesis features, and the development of all manner of immune response. Today, the late phases of preclinical trials that aim to assess the efficacy and safety of antiviral vaccines are mainly conducted using rhesus macaques (*Macaca mulatta*), crab-eating macaques (*M. fascicularis*), and green monkeys (*Chlorocebus sabaeus*). However, long-term maintenance of a significant number of large primates in experimental laboratories faces ethical restrictions and is extremely expensive, while primates kept in outdoor nurseries need to undergo long-term acclimatization and examination to exclude any background pathol ogy before they can be used in experimental work. Furthermore, macaques have a much higher variability of major histocompatibility complex (MHC) class I genes compared to humans; so, in some cases the animals either need to be genotyped before inclusion in experiments or the number of animals per study group needs to be significantly increased, which further raises the cost of the trials [1].

Common marmosets (*Callithrix jacchus*, CM) are used in many areas of biomedical research, including reproductive biology, cognitive research, autoimmune and infectious diseases, oncology, and toxicology [2].* C. jacchus *cells are also used in embryology and regenerative medicine [3].

A number of characteristics of CMs as a biological species make them a valuable laboratory model. These characteristics include: (1) phylogenetic proximity to humans; (2) small body weight (300–500 g); and (3) the relative ease of laboratory breeding and maintenance [4]. An important feature of *C. jacchus *is the minimal diversity of both MHC class I and class II gene loci [5, 6], which contributes to highly reproducible study results.

CMs are susceptible to many viral, protozoan, and bacterial human pathogens [7], including the yellow fever virus, Epstein–Barr virus and other herpesviruses, hepatitis A virus, Junín virus, measles virus, hepatitis E virus, etc. Working with pathogens using CMs poses much fewer technical challenges, and is, therefore, associated with reduced risks for the personnel, than working with large primates. Furthermore, the genome of CMs has been fully sequenced; so, these primates can be adequately used in* in vivo *trials of novel gene therapy products, including experiments requiring transgenic animals [8, 9].

In combination with the recently elaborated procedures of assessment of the parameters of humoral and T cell-mediated immunity [10], the aforementioned factors make *C. jacchus *an optimal nonhuman primate species for the preclinical trials of safety, immunogenicity, and protectivity of antiviral vaccines. Nevertheless, broader experimental use of CMs requires solving a number of problems, including the development and standardization of laboratory husbandry protocols, as well as the functional and morphological characterization of the organs of their immune system.

In this study, we optimized the conditions of longterm laboratory breeding and maintenance of CMs, established the reference values of the main complete blood count (CBC) and serum chemistry parameters evaluated during preclinical trials of antiviral vaccines, and described the histological characteristics of the lymphoid organs of laboratory-bred CMs during the development of post-vaccination immune response.

## METHODS

**Ethics statement**

The protocols of all the experiments involving primates described in this study were approved by the Ethics Committee of the Chumakov Federal Scientific Center for Research and Development of Immunobiological Products (protocols No. 110520-1 dated May 11, 2020, No. 140720-1 dated July 14, 2020, and No. 141021-2 dated October 14, 2021).

**Laboratory breeding and maintenance of common marmosets**

The animals were kept at the Laboratory of modeling of immunobiological processes with the experimental clinic of Callitrichidae of the Chumakov Federal Scientific Center for Research and Development of Immune-and-Biological Products of the Russian Academy of Sciences (Laboratory), in compliance with Sanitary Regulations 3.3686-21 “Sanitary and Epidemiological Requirements for Preventing Infectious Diseases,” State Standard GOST 33218- 2014 “Guidelines for Accommodation and Care of Laboratory Animals,” and the Directive of the European Parliament and the Council of the European Union 2010/63/EC dated September 22, 2010.

The Laboratory facilities included the breeding zone and the experimental zone, with separated personnel and material flows. The automatic ventilation and air conditioning system ensured a year-round air temperature of 24–30°C and ≥50% humidity; it contained two independent circuits for the breeding zone and the experimental zone.

The rooms of the breeding zone had windows for natural daylight, as well as daylight lamps that were switched on daily in the time interval between 7.00 a.m. and 5.00 p.m. all year round.

In the breeding zone, CMs were housed in family groups in enclosures sized 810 × 470 × 1760 mm (L × D × H). The family groups of CMs consisted of an adult animal pair and two generations of their offspring. The total number of animals per enclosure in the breeding zone ran up to six. At the age of 10– 13 months, the offspring were placed into separate enclosures for immature animals. New family pairs were formed of primates aged at least 18 months; they were subsequently monitored to assess the individual compatibility of the new pair.

The daily energy value of the diet used in the Laboratory was 140 kcal per adult animal weighing 350–450 g; 18–24% of the diet consisted of protein from boiled chicken meat, baked cottage cheese, eggs, buckwheat and oatmeal porridge. The diet was daily supplemented with 360 IU of vitamin D3, calcium gluconate, and a multivitamin complex.

Autoclavable dispensers (volume, 100 mL) of drinking water meeting the State Standard GOST R 51232-98 were mounted at the upper level of the walls of each enclosure.

Food leftovers were removed from the enclosure trays daily before morning feeding and after 12 p.m. The biological waste in the experimental zone was decontaminated by autoclaving.

**Experimental manipulations with common marmosets**

All the manipulations involving CMs were conducted by certified veterinarians or by researchers certified by the Federation of European Laboratory Animal Science Associations (FELASA) and trained to work with nonhuman primates.

Experimental procedures were performed in a microbiological safety cabinet class II VIS-A-VIS, type A, installed in an operating room equipped to perform all the needed procedures with CMs, including biological material sampling, the administration of experimental preparations, and surgical interventions.

The animals were subjected to inhalational general anesthesia supplied via a full-face mask using the 410AP anesthesia machine (Univentor, Malta) with an air–gas mixture containing 4% isoflurane for anesthesia induction and 2–2.5% isoflurane for maintenance of anesthesia.

Subcutaneous radio chips of ISO 11784 standard (LifeChip, Destron Fearing, USA) in capsules made of biocompatible glass with an anti-migration coating were used for animal identification. The microchip is a passive device without a power source, so it can be used throughout the entire length of the life of an animal.

The body weight of the primates was measured using a Pioneer PA4102 electronic balance (Ohaus, USA).

All experimental manipulations with the primates were performed in the operating room, excluding any visual or auditory contact with other animals.

**Complete blood count and serum chemistry**

Whole blood samples for CBC and serum chemistry were collected by puncturing the femoral vein using 2.5 mL three-part syringes with 27G needles. The maximum blood volume sampled in a single procedure was under 3 mL (≤ 8% of the circulating blood volume). For CBC, the syringes were prefilled with a Na- EDTA solution (final concentration, 5 mmol Na-EDTA per liter of blood). For the serum chemistry analysis, blood samples were collected into dry sterile test tubes and mixed and incubated at room temperature for 45 min; the serum was then separated by 10-min centrifugation (5810R, Eppendorf, Germany) at 600 g.

CBC with erythrocyte and leukocyte counts, as well as the leukocyte differential count, was carried out in a Goryaev chamber using Romanowsky staining.

The CM serum chemistry analysis was performed on a Cobas c111 automated analyzer (Roche, Switzerland) using the respective reagent kits. Values below the limit of detection of the instrument were counted as 0.

**Histological analysis of post-vaccination changes in the lymphoid organs**

Seven animals (three males and four females) aged 2–5 years, born in the Laboratory and included in preclinical trials of the inactivated whole-virion purified adsorbed vaccine against COVID-19 CoviVac, were used to study post-vaccination changes in the lymphoid organs of CMs [11].

On the day of the first immunization and 14 days later, 250 μL of the vaccine preparation (a suspension for intramuscular injection) were injected into the thigh muscles of the right and left legs of the animals in the experimental group (total injected volume, 500 μL per animal). The animals in the control group were injected with an identical volume of placebo containing the vaccine adjuvant (aluminum hydroxide) via the same route on the same days.

The animals were euthanized by anesthesia overdose (intramuscular injection of a threefold dose of a mixture of Xyla (De Adelaar, Netherlands) and Zoletil (Virbac, France) under isoflurane anesthesia.

Lymphoid organs (thymus, spleen, mesenteric lymph node, and inguinal lymph node draining the injection site) for histological examination were fixed immediately after necropsy by submersion in 10% buffered formalin (Biovitrum, Russia).

The organ samples were subjected to automated histological processing, which involved sequential dehydration in increasing concentrations of ethanol and xylene, embedding into Histomix paraffin medium (Biovitrum) on a Leica EG1150H paraffin embedding station (Leica, Germany), and microtomy of the resulting blocks with embedded samples on a Leica RM 2245 rotary microtome (Leica) to obtain 3 μm thick paraffin sections. The sections of lymphoid organs were mounted onto microscope slides, dried, deparaffinized, hydrated, stained with alum hematoxylin and water–lcohol eosin (Biovitrum), and placed under coverslips in a BioMount medium (Bio-Optica, Italy) to obtain stable histological specimens. One to four representative sections of proper quality were obtained per block.

The prepared sections were analyzed under a Zeiss Axio Observer A1 optical microscope (Carl Zeiss, Germany). Representative microimages were obtained using an AxioCam 305 high-resolution digital microscopy camera in the Zeiss Zen 2 lite blue edition software (Carl Zeiss). Microimage processing and panel compilation was performed using the AxioVision v.3.0 (Carl Zeiss) and GIMP (S. Kimball, P. Mattis, USA) software.

**Statistical analysis**

The age of the females at the time of first litter delivery, the survival rate of offspring during the neonatal period, and interdelivery intervals are presented as mean values and the standard deviation (SD). The statistical significance of differences in the parameters of CBC and serum chemistry was assessed using the Mann–Whitney test in the GraphPad Prism 9 (9.3.0) software. Differences were considered significant at *p * < 0.05.

## RESULTS

**Laboratory breeding of common marmosets**

The retrospective study was based on the data obtained by observing 23 female CMs born in the Laboratory, which delivered a total of 69 litters during the period between 2015 and 2023. *Figure* shows the estimated mean age of females at the first delivery, as well as the mean interdelivery interval.

**Fig. 1:**
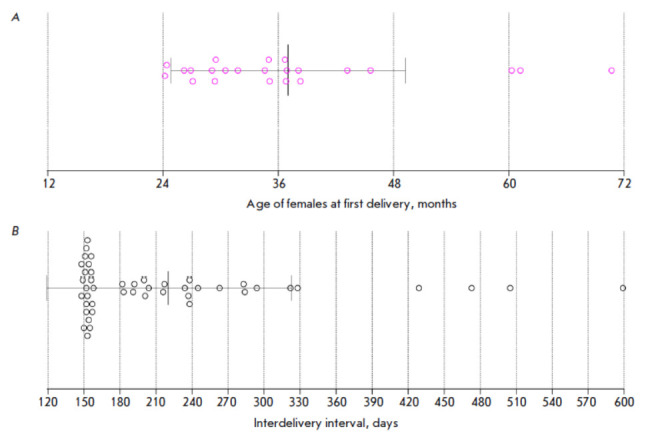
Frequency of deliveries in female laboratory-bred common marmosets. (*A*) Pink circles indicate the age of females (*n *= 23) at the time of first delivery. (*B*) Black circles indicate interdelivery intervals (*n *= 46). Vertical solid lines indicate the mean value and standard deviation

The mean age of female CMs at the first delivery was 37 months (SD = 12.2); the minimal age was 24.2 months (*Fig. 1A*).

The mean interdelivery interval during the observation period was 220.1 days (SD = 102.9); 21 out of 46 litters were delivered 148–158 days after the previous delivery (*Fig. 1B*). Since the average gestation period in CMs is 143–144 days [4], the observed 148–158-day interdelivery interval meant that the next conception occurred within one or two weeks post-partum.

During the study period, a total of seven of the 23 observed females delivered one litter; four females delivered two litters; eight females, three litters; two females, four litters; one female delivered eight litters; and one female, 14 litters. All the newborn CMs that had survived the neonatal period were considered survivors, since no mortality was observed after 28 days of life. Gastrointestinal disorders during the first three days of life were the predominant cause of death. In the subsequent analysis, the infants that had died during the neonatal period were accounted as stillborn. Hence, the mean number of surviving offspring per delivery during the observation period was 1.45, with significant variation between individual females.

Throughout the observation period, the most common delivery outcome (*N *= 69) in laboratory-bred CM females was giving birth to two infants (31/69). In 20/69 cases, there was one living newborn; and in 6/69 cases, three newborns. In 12 cases, CM females delivered one to five infants that were stillborn or died within the first three days of life.

According to our observations, there were no significant changes in female fertility until at least the eighth to ninth delivery, but this conclusion needs further verification, since only two females out of 23 delivered more than four litters during the observation period.

**Fig. 2:**
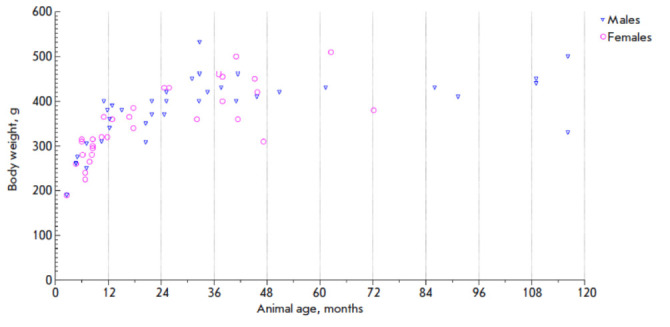
Body weight and age of laboratory-bred common marmosets. The total number of animals is 69 (37 males and 32 females)

In April 2023, 69 laboratory-bred animals (37 males and 32 females aged from 2.6 months to 9.6 years) were weighed within a one-week period. The results are summarized in *Fig. 2*.

**Fig. 3:**
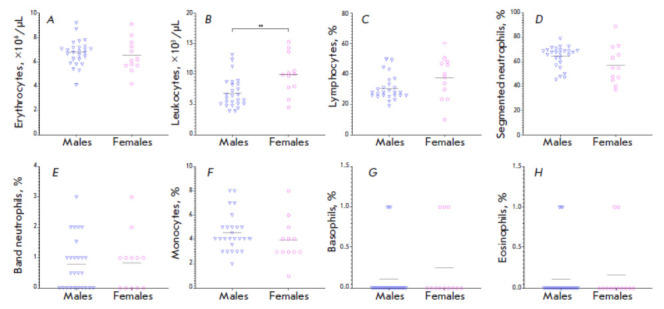
Counts of (*A*) erythrocytes, (*B*) leukocytes, and the percentage of (*C*) lymphocytes, (*D*) segmented neutrophils, (*E*) band neutrophils, (*F*) monocytes, (*G*) basophils and (*H*) eosinophils in the leukocyte differential in the blood of laboratory-bred common marmosets (*N *= 38). Horizontal lines show the mean value. The statistical significance of the differences in the studied parameters between males and females was assessed using the Mann–Whitney test. **statistically significant differences (*p* < 0.05)

The body weight of CMs increased rapidly during their first 1.5 years of life. By the age of 18–20 months, the mean body weight of the animals had reached 400 g and stayed at the same level in all studied CMs aged up to 9.6 years. No significant differences in body weight were detected between the males and females (Mann–Whitney test, *p *= 0.0823).** Determining the reference values of the parameters of complete blood count and serum chemistry** In order to determine the reference values for CBC, blood samples were collected from a total of 38 CMs (26 males and 12 females) aged 2–5 years over the period from May 2020 to December 2021. The CBC results for laboratory-bred CMs are summarized in *Fig. 3*.

The mean erythrocyte count in the blood of laboratory- bred CMs was 6.6 (4.1–9.2) ×10^6^ cells/μl; the mean leukocyte count was 7.8 (3.9–5.3) ×10^3^ cells/ μl. In the leukocyte differential, the mean percentage of lymphocytes was 32.8 (10–0)%; segmented neutrophils, 61.8 (37–9)%; band neutrophils, 0.8 (0–)%; monocytes, 4.3 (1–)%; basophils, 0.2 (0–)%; and eosinophils, 0.1 (0–)%. Females had a higher mean leukocyte count (Mann–hitney test, *p *= 0.0047) compared to males. No statistically significant differences in other hematological parameters were detected between males and females.

**Fig. 4:**
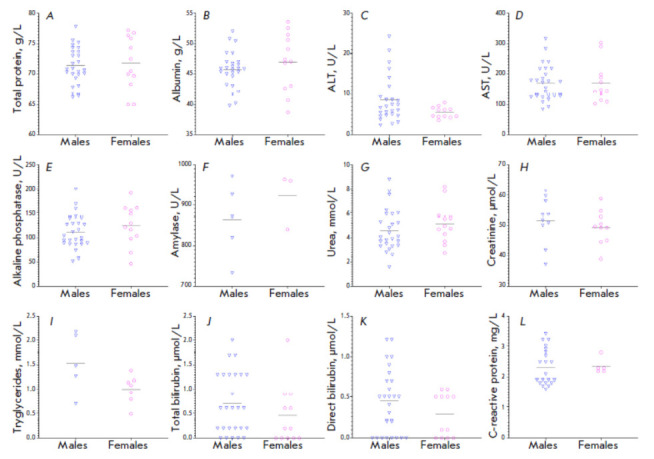
Serum chemistry parameters in laboratory-bred common marmosets. (*A*) Total protein level; (*B*) albumin level; (*C*) ALT activity; (*D*) AST activity; (*E*) alkaline phosphatase activity; (*F*) amylase activity; (*G*) urea level; (*H*) creatinine level; (*I*) triglyceride level; (*J*) total bilirubin level; (*K*) direct bilirubin level; and (*L*) C-reactive protein level are presented. For creatinine, *N *= 20; for triglycerides, *N *= 12; for amylase, *N *= 8; for C-reactive protein, *N *= 26; and for other parameters, *N *= 38. Horizontal lines represent the mean value. The statistical significance of the differences in the studied parameters between males and females was assessed using the Mann–Whitney test

In order to determine the reference values of the parameters of serum chemistry in laboratory-bred CMs, the creatinine level was measured in 20 animals (10 males and 10 females); the level of triglycerides, in 12 animals (five males and seven females); amylase activity, in eight animals (five males and three females); C-reactive protein level, in 26 animals (21 males and five females); and other parameters were measured in 38 animals (26 males and 12 females). *Figure 4* shows the serum chemistry data for the laboratory-bred animals.

The mean serum level of total protein in laboratory- bred CMs was 71.3 (65–77.8) g/L; albumin level, 44.8 (38.7–53.58) g/L; ALT activity, 8.0 (2.4–24.3) U/L; AST activity, 182.8 (84.3–316.1) U/L; alkaline phosphatase activity, 106.5 (46.7–199) U/L; amylase activity, 885.7 (732.9–964) U/L; urea level, 4.8 (1.6–8.8) mmol/L; creatinine level, 51 (37.3–61.4) µmol/L; triglycerides level, 1.22 (0.48–.17) mmol/L; total bilirubin level, 0.8 (0–) μmol/L; direct bilirubin level, 0.4 (0–.2) μmol/L; and C-reactive protein level, 2.3 (1.6–.4) mg/L. No statistically significant differences in serum chemistry parameters were revealed between males and females (Mann–hitney test,* p *> 0.05 for all the parameters).

**Post-vaccination changes in the lymphoid organs of common marmosets**

A histological analysis of the main lymphoid organs in four vaccinated (one male and three female) and three control (two male and one female) CMs aged 2–5 years was conducted during preclinical trials of the inactivated purified whole-virion adsorbed vaccine against COVID-19 CoviVac. We characterized the morphological structure of lymphoid organs in the animals that received a placebo and described the microstructural changes in the thymus, spleen, and lymph nodes observed during the development of the specific post-vaccination immune response.

**Fig. 5:**
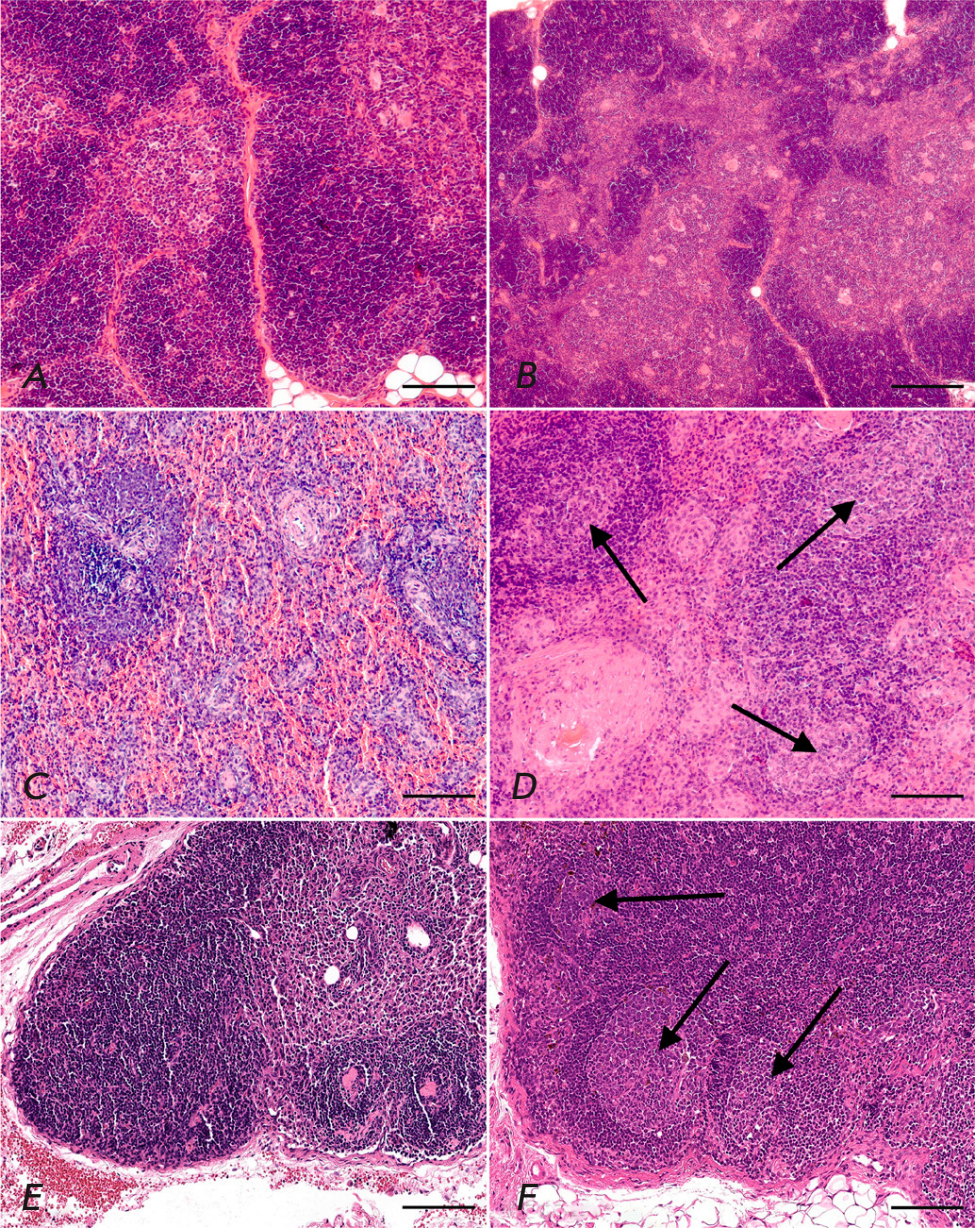
Lymphoid organs of common marmosets immunized with a whole-virion inactivated vaccine CoviVac (*B*, *D*, and *E*) compared with the animals who received the placebo (*A*, *C*, and *E*). The morphology of the following lymphoid organs is presented: (*A*, *B*) – thymus; (*C*, *D*) – spleen; (*E*, *F*) – the regional (inguinal) lymph node. Arrows show the germinal centers in the cortical plateau of the lymph node and in the mantle zone of the white pulp of the spleen, the region of T-dependent B-immunogenesis. Hematoxylin and eosin, ×100 magnification. Scale bar, 200 μm

*The morphology of lymphoid organs in the control animals. *The thymus (*Fig. 5A*) was preserved in all the animals. The lighter colored medullary and darker cortical substance of the organ were easily distinguishable morphologically. Accidental (stress-induced) involution of the cortical substance of the thymus, as well as lipomatosis of the cortical substance, was either absent or minimal. Histologically, the organ structure corresponded to what are normal observations for this species described in the literature [12] (including the presence of Hassall’s corpuscles of the medulla).

The spleen (*Fig. 5C*) of the control primates had a proper white and red pulp structure: there were neither atrophic nor dystrophic changes, as well as no pathologic enlargement of the white pulp zones; red pulp was moderately congested. Spleen macrophages in the control animals were not vacuolated and did not show visible accumulation of the adjuvant components (aluminum hydroxide gel) or other substances. In all the animals studied, no morphological signs of myeloid metaplasia of red pulp were revealed.

In the control animals, the regional inguinal lymph node draining the placebo injection site (*Fig. 5E*) had a proper structure and consisted of the cortical plateau, the paracortical region with medullary cords, and the sinus system. In all the studied animals, the lymph node had no pathological changes and morphologically corresponded to the normal observations for the species.

The mesenteric lymph node (not shown) in both vaccinated and control animals had no distinctive features or pathological changes. Morphological manifestations of immunogenesis were observed: strongly marked germinal (light-colored) centers in the cortical plateau and minimal histiocytosis of the marginal sinus, which normally represents the function of the organ constantly undergoing antigenic stimulation from the intestine.

*The morphology of the lymphoid organs of the vaccinated animals*. No morphological differences were observed between the thymus of vaccinated animals (*Fig. 5B*) and those that had received the placebo. The thymus is a primary lymphoid organ where antigen- mediated B-immunogenesis does not elicit morphofunctional changes.

Formation of germinal (light-colored) centers in the mantle zone of the white pulp was detected in some spleen samples harvested from vaccinated CMs (*Fig. 5D*). This pattern morphologically indicates a T-dependent B-immunogenesis, corresponding to the development of a post-vaccination response. Otherwise, the spleen structure was identical to that in the control animals. We did not detect any vacuolization or visible accumulation of vaccine components, aluminum hydroxide gel, or other substances in the macrophages or within the marginal zone and the splenic red pulp of the vaccinated animals.

In vaccinated CMs, the regional inguinal lymph node draining the vaccine injection site (*Fig. 5F*) had a proper structure and consisted of the cortical plateau, the paracortical region with medullary cords, and the sinus system. Morphofunctional manifestations of immunogenesis of different intensities were observed: the emergence of germinal (light-colored) centers in the cortical plateau (the so-called B-dependent zone of the lymph node), as well as minimal histiocytosis of the marginal sinus, which corresponds to the development of a post-vaccination response and is morphologically similar to the events occurring in human lymph nodes upon antigen exposure.

## DISCUSSION

Common marmoset *(C. jacchus*) is a nonhuman primate species endemic to the tropical Atlantic coastal zone in the northeastern regions of Brazil. In the wild, CMs live in families consisting of a stable pair of adult animals and their numerous offspring. In groups, one female is socially dominant, suppressing the reproductive activity of other females (in particular, mothers tend to dominate over daughters) [4]. CMs are diurnal and live in the dense upper and middle deciduous canopies, hiding from snakes and birds of prey.

The ethological needs of the species were taken into account for the development of techniques for the long-term laboratory maintenance of the CMs: the day/night light cycle in the animal breeding zone corresponds to daylight hours in the natural habitat; the structure of family groups matches that in the wild; and high enclosures allow the animals to move to the upper sections (i.e., to implement a behavioral cascade associated with searching for shelter when threatened). Changing of the arrangement of environmental enrichment elements inside the enclosures was performed by a veterinarian, in accordance with a cyclical scheme. Environmental enrichment elements (bells, mirrors, branches, hangers, swings, hammocks, and bars) aimed to extend the spectrum of behaviors and motion patterns and make foraging activity more challenging (feeders with drilled holes arranged in different areas within the enclosures). Primates get used to the unchanging environmental enrichment elements and lose interest within 3–5 days, which may subsequently cause stereotypy or elevated aggression within the group.

In this study, we determined the mean age of the females at the first litter delivery, the mean interdelivery interval, the survival rate of infants, and the kinetics of body weight gain in laboratory-bred CMs. Altogether, these findings allow one to manage the colony population depending on the experimental needs. According to our data, a CM female on average gives birth to about three babies per year and the population of laboratory-bred CM colonies can be increased by both maximizing the number of pairs and choosing the most fertile females.

Safety assessment of immunobiological products using laboratory-bred CMs requires the identification of the reference values of the parameters of CBC and serum chemistry, since the published reference values are often based on results obtained by studying the biomaterial collected from a small number of animals housed in outdoor enclosures in nurseries and zoos. An analysis of the samples collected from 38 healthy male and female CMs aged 2–5 years revealed the main parameters of CBC and serum chemistry. Statistically significant differences between males and females were observed only for the leukocyte count (Mann–Whitney test, *p *= 0.0047).

During the preclinical assessment of vaccine safety and immunogenicity, it is important to characterize the immunization-induced histological changes in the lymphoid organs. In this study, we performed a histological analysis of the lymphoid organs of CMs after the administration of an inactivated whole-virion adsorbed vaccine against COVID-19 and in the placebotreated group.

The histological examination revealed Hassall’s corpuscles (clusters of concentric eosinophilic terminally differentiated epithelial cells) in the thymic medulla of the CMs, which makes their thymus morphologically similar to the human thymus. It is known that in rodents, which are most frequently used in preclinical trials of immunobiological products, including vaccines, the thymic structure differs from that of humans and their thymic medulla contains no Hassall’s corpuscles [13].

Another important histologic finding was that the studied primates had no myeloid metaplasia of the splenic red pulp when the morphological signs of hematopoietic tissue occurred outside the bone marrow. Like in humans, myeloid metaplasia in CMs is regarded as a background pathological condition associated with bleeding [14]: so it is easier to classify changes in the spleen of CMs and extrapolate them to humans, as compared to the data obtained when working with rodent spleen.

Therefore, in this study, we have garnered evidence that there is high similarity between the structure of the lymphoid organs of CMs and humans both in the control animals and during the development of a post-vaccination immune response.

Our findings suggest that CMs bred under isolated laboratory conditions preventing any background infectious pathology are an adequate laboratory model for characterizing the safety and immunogenicity profiles of antiviral vaccines in preclinical trials with a high level of confidence.

## CONCLUSIONS

Owing to a number of biological features particular to the species, as well as the development of procedures of breeding, long-term maintenance, and experimental management, laboratory-bred CMs have recently been used in a number of biomedical studies, including preclinical trials of inactivated [11, 15] and adenoviral vector-based [10] vaccines against COVID-19, an adenoviral vector-based vaccine against Middle East respiratory syndrome-related coronavirus [16], and a candidate recombinant vaccine against hepatitis E [17].
